# The influence of intraocular pressure in the onset and progression of myopia in children and adolescents: a review of mechanisms and clinical significance

**DOI:** 10.3389/fmed.2026.1836275

**Published:** 2026-05-08

**Authors:** Weihai Xu, Qiliang Li, Yanchun Guo

**Affiliations:** Binhai County People's Hospital, Yancheng, China

**Keywords:** axial length, biomechanics, children and adolescents, intraocular pressure, myopia, myopia control

## Abstract

Intraocular pressure (IOP), the fluid pressure inside the eye, is gaining attention for its possible involvement in the onset and advancement of myopia among younger populations. This systematic assesses the biomechanical impact of IOP on ocular tissues, including the retina and choroid, and its connection to critical pathological changes like axial elongation and scleral structural alterations. By evaluating findings from contemporary cross-sectional analyses, longitudinal cohort studies, and interventional trials, this article examines the epidemiological relationships between IOP levels and both the incidence and progression rate of myopia. Additionally, it discusses the potential clinical significance of incorporating IOP assessment into myopia management approaches. Through a synthesis of multimodal ocular biometric data, this review seeks to present an updated viewpoint on the intricate pathophysiology of myopia and to establish a foundation for future clinical evaluation and therapeutic strategies.

## 1 Introduction

Myopia, particularly in children and adolescents, has emerged as a significant global public health concern, with its pathological core being excessive axial elongation. For a long time, the role of intraocular pressure (IOP) in ocular development and refractive status has attracted attention. A classic hypothesis suggests that higher eye pressure may mechanically stretch the posterior sclera of the eyeball, leading to axial length elongation and thereby inducing or accelerating myopia progression (1, 2). This hypothesis is supported by some clinical observations, such as in adults, myopia wearing, especially high myopia, is often associated with higher eye pressure, and the risk of open-angle glaucoma is significantly increased in myopic populations (2, 3). However, this seemingly intuitive relationship is highly controversial in children and adolescents with myopia. Early studies, such as the Cross-sectional survey by Quinn et al. (4), found that myopic children appeared to have higher eye pressure than non-myopic children. However, subsequent large-scale, more rigorously designed prospective cohort studies, such as the Anyang Childhood Eye Study (5) and the Handan Offspring Myopia Study (6), reached entirely different conclusions, suggesting that eye pressure is not associated with the rate of myopia progression in children. These conflicting results have prompted profound rethinking within the academic community regarding the role of eye pressure. For instance, a cross-sectional study comparing children and adolescents in high-altitude Tibet with those in low-altitude Chongqing found that the Tibetan group, which exhibited a lower prevalence of myopia and slower progression rates, also presented with significantly lower IOP (7). This association implies that environmental or physiological factors associated with high altitude, potentially including lower IOP, may contribute to the observed differences in myopia prevalence and axial length growth. Furthermore, while myopic eyes have been observed to have slightly higher IOP compared to emmetropic and hyperopic eyes, this difference may not be clinically significant. Importantly, longitudinal data from a cohort of rural Chinese children indicated that baseline IOP was not associated with the subsequent development of myopia, its progression rate, or axial length elongation over a 3.5-year follow-up period (6). This suggests that static IOP measurements alone may not be a strong predictive factor for myopia progression in all populations. However, the relationship appears more complex in specific contexts. In children with non-pathological high myopia, higher IOP was paradoxically associated with slower axial growth in a longitudinal analysis, a finding potentially confounded by the influence of ocular compliance on IOP measurement (8). Conversely, experimental studies in young adults have demonstrated that a transient increase in IOP can induce temporary axial elongation and subfoveal choroidal thinning, with the axial changes largely attributable to choroidal thickness variations (9). This highlights the dynamic and potentially transient influence IOP may exert on ocular dimensions. The role of IOP may also vary with the degree of myopia and the presence of pathological changes. In eyes with high myopia, IOP was not found to be significantly associated with the presence or progression of myopic macular degeneration or posterior staphyloma in non-glaucomatous eyes (10). Nevertheless, the use of antiglaucoma medications was independently associated with reduced axial elongation in highly myopic eyes, suggesting a potential for pharmacologic modulation of eye growth independent of IOP-lowering effects (10). This is supported by animal studies where topical IOP-lowering medication (carteolol) did not significantly affect the magnitude of lens-induced axial elongation in young guinea pigs, indicating that physiological IOP variations may not play a major role in experimental myopization in this model (11). Collectively, these findings underscore that the epidemiological relationship between IOP and myopia is not straightforward and may be influenced by age, baseline refractive error, ocular biomechanics, and measurement context, warranting a nuanced interpretation of its clinical significance.

Current research suggests that the relationship between intraocular pressure and myopia may be indirect, involving modulation of posterior segment biomechanics, vascular perfusion, and optical signaling. One primary pathway is through its effect on the biomechanical properties of the sclera. IOP represents a continuous distending force on the ocular coat. In experimental myopia, scleral remodeling, characterized by reduced thickness and altered extracellular matrix composition, is a key event facilitating axial elongation. The sclera’s biomechanical response to IOP is therefore critical. Studies have shown that anterior scleral thickness in myopic eyes is associated with several ocular parameters, including axial length and IOP, with longer axial length correlating with scleral thinning at specific meridians (12). The biomechanical properties of the cornea, which may reflect broader ocular biomechanics, are also associated with myopia. In both children and adults, a larger corneal deformation amplitude (indicating a more deformable cornea) is significantly associated with longer axial length, steeper corneal curvature, older age, and lower IOP (13). This interplay between IOP and scleral biomechanics suggests that IOP fluctuations, particularly those induced by accommodation, could contribute to a biomechanical susceptibility that promotes scleral remodeling and elongation (2). A second potential mechanism involves choroidal blood flow. The choroid, positioned between the sclera and retina, is highly vascular and may act as a biomechanical or metabolic buffer. Transient IOP elevation has been shown to cause immediate subfoveal choroidal thinning (9). Since choroidal thickness is negatively correlated with axial length in myopic children (14). IOP-induced choroidal thinning could indirectly influence scleral metabolism and growth signaling. Lowering IOP is hypothesized to increase choroidal blood perfusion, potentially alleviating scleral hypoxia—a driver of scleral remodeling—and thereby slowing axial elongation, especially in pathologic myopia (11).

The core objective of this review is to systematically review and analyze the existing evidence on the relationship between intraocular pressure and myopia progression. Through this critical examination, the review aims to provide directional guidance for future research in this field and offer scientific evidence for clinical practice decisions regarding intraocular pressure management in myopia prevention and control.

## 2 Epidemiological and cross-sectional evidence on the association between intraocular pressure and myopia in children and adolescents

### 2.1 Differences in IOP levels and myopia prevalence among different populations

Comparative studies between populations in distinct geographical and environmental settings provide valuable insights into the potential role of intraocular pressure (IOP) in myopia development. A cross-sectional study comparing children and adolescents in Chongqing (low altitude) and Tibet (high altitude) in China revealed significant differences in ocular biometric parameters and myopia prevalence (7). The Tibet group exhibited a lower prevalence of myopia (39.7% vs. 47.6%), a less myopic spherical equivalent, and a significantly shorter axial length compared to the Chongqing group. Crucially, the average IOP was also significantly lower in the Tibetan children. Further analysis showed that the differences in myopia prevalence and spherical equivalent between the two regions became more pronounced in older age groups (12–14 and 15–18 years), while the differences in axial length and the axial length to corneal radius ratio (AL/CR) were evident across all age subgroups. The study identified axial length as the primary contributor to the refractive difference, but also noted that IOP was one of the several ocular biometric parameters that differed, alongside corneal thickness, white-to-white distance, and anterior chamber depth. The slower rate of myopia progression and axial elongation observed in the Tibetan children, coupled with their lower IOP, suggests that environmental factors prevalent at high altitudes—such as higher ultraviolet radiation exposure—may influence ocular development. These factors could potentially modulate IOP or other physiological pathways, thereby contributing to a protective effect against myopia progression.

### 2.2 Correlation between IOP and myopia severity and ocular biometric parameters

Investigating the relationship between intraocular pressure (IOP) and the severity or progression of myopia has yielded complex and sometimes contradictory findings, highlighting the need to consider IOP as a potential confounder in structural analyses. In a cohort of rural Chinese children, while myopic eyes had a slightly higher baseline IOP compared to emmetropic and hyperopic eyes, this difference was not clinically significant (6). More importantly, longitudinal analysis over 3.5 years found no association between baseline IOP and the subsequent development of myopia, its rate of progression, or axial length elongation. This suggests that in general pediatric populations, IOP may not be a primary driver of myopia onset or progression. However, the relationship appears different in specific subgroups. A study focusing on children with non-pathological high myopia found that higher IOP was associated with slower, not faster, axial growth over time (8). The authors proposed that this counterintuitive finding might be confounded by ocular compliance, where a more compliant (less rigid) eye in a growing child could manifest a lower measured IOP despite greater susceptibility to elongation. This hypothesis underscores the complexity of interpreting IOP measurements in the dynamic, developing pediatric eye. In large-scale, high-quality registries like the Shanghai Child and Adolescent Large-scale Eye Study (SCALE-HM), IOP is systematically measured as a routine parameter in children with high myopia (15). Although the direct association between IOP and specific high myopia-related fundus lesions (like peripapillary atrophy or diffuse chorioretinal atrophy) was not the primary focus of the initial methodology report, the comprehensive data collection establishes a critical foundation for future analyses to elucidate IOP’s role in pathological myopia development. Furthermore, when assessing structural changes associated with myopia, such as alterations in the optic disc or retinal nerve fiber layer (RNFL), studies appropriately treat IOP as a key covariate. For instance, research evaluating optic disc parameters and circumpapillary RNFL thickness in children with refractive errors using swept-source OCT employed multivariable linear regression models that controlled for IOP, among other factors like axial length and keratometry (16). This analytical approach is essential because IOP is a known determinant of optic disc morphology and can influence RNFL thickness measurements. Failing to account for IOP could lead to erroneous conclusions about the structural impact of myopia itself. Therefore, while IOP may not consistently show a direct, simple correlation with myopia progression rate in all studies, its consideration is paramount in multivariate models analyzing myopia-associated anatomical changes to isolate the specific effects of axial elongation from those potentially related to pressure.

## 3 Intraocular pressure with myopia development

### 3.1 Biomechanical effects of intraocular pressure on the retino-choroid-scleral complex

Intraocular pressure (IOP) exerts a steady-state stress on the ocular wall, particularly on the posterior sclera. During the developmental period, sustained IOP may promote scleral thinning and extension by influencing the activity of scleral fibroblasts and the remodeling of the extracellular matrix, thereby driving axial elongation. Biomechanical studies demonstrate that the strain response of the sclera, choroid, and retina is directly related to the magnitude of IOP change (17). In glaucoma patients, IOP lowering induces measurable strain in these posterior tissues, with the sclera showing a positive radial strain response, contrasting with the negative strain observed in anterior optic nerve head tissues (17). This indicates that IOP-generated stress is transmitted through the ocular coats. The biomechanical properties of the posterior eye, including the sclera and the Bruch’s membrane-choroid complex (BMCC), are crucial for understanding disease pathogenesis (18). Research indicates that the stiffness of the BMCC increases with strain and is closely linked to aging and related diseases (18). In the context of myopia, the sclera’s mechanical behavior is fundamental. Experimental models show that interventions like scleral cross-linking (SXL), which increases scleral stiffness, can slow axial elongation in myopic animal models without significantly altering IOP (19). Computational models further support that local scleral weakening, even under normal IOP, can trigger posterior staphyloma formation through growth and remodeling processes, highlighting the role of scleral biomechanical integrity in resisting deformation (20). The relationship between IOP and ocular rigidity (OR), a key biomechanical property, is also evident. Studies show an inverse correlation between pulsatile choroidal volume change (ΔV) and ocular rigidity, suggesting that a more rigid eye exerts greater resistance to choroidal expansion driven by pulsatile blood flow (21). This interplay between IOP, scleral stiffness, and choroidal dynamics forms a complex biomechanical environment. Choroidal thickness, which reflects intraocular hemodynamics and biomechanical conditions, is negatively correlated with axial length. IOP may indirectly regulate choroidal thickness by affecting the perfusion pressure of the choriocapillaris (18). Thinning of the choroid is considered a signal of myopia progression. Finite element analyses of the optic nerve head (ONH) reveal that morphological parameters like lamina cribrosa depth and choroidal thickness are predictors of biomechanical strain under IOP, linking structure to mechanical response (22). In summary, IOP acts as a persistent biomechanical force on the retino-choroid-scleral complex. During ocular growth, it can influence scleral extracellular matrix remodeling, potentially leading to compromised stiffness and axial elongation. Concurrently, IOP interacts with choroidal hemodynamics, affecting its thickness. The biomechanical coupling between IOP, scleral properties, and choroidal behavior is therefore a critical pathway in the development and progression of myopia.

### 3.2 Interaction between intraocular pressure and peripheral retinal defocus

The pattern of peripheral retinal defocus is a crucial visual signal regulating axial growth. Research indicates that most myopic adolescents exhibit peripheral hyperopic defocus. The biomechanical properties of the cornea and sclera, which are influenced by IOP, are integral to overall ocular shape and rigidity (18). Studies on corneal biomechanics in varying degrees of myopia show that several parameters indicative of corneal stiffness progressively decrease with increasing myopia and axial length, independent of IOP and central corneal thickness (23). This suggests a systemic change in ocular coat biomechanics in myopia, which could affect how the eye responds to both IOP and visual signals. The deformation of the posterior pole under biomechanical stress is a key consideration. Finite element modeling suggests that during large eye movements, the peripapillary Bruch’s membrane may wrinkle, forming undulations at its interface with the retina and choroid (24). Such micro-scale morphological alterations, potentially influenced by the IOP-strain environment, could theoretically perturb the precise optical path for peripheral retinal images. Furthermore, the interaction between IOP and eye shape is evident in conditions like glaucoma and high myopia. Longitudinal studies in children with large vertical cup-to-disc ratios show that progressive optic disc tilt during myopia progression is associated with a higher risk of developing retinal nerve fiber layer defects (25). This morphological alteration of the optic nerve head, driven by axial elongation (a process linked to defocus), occurs within an IOP-loaded biomechanical environment. The deformation of the optic nerve head under IOP is a well-studied phenomenon in glaucoma (22). While these studies focus on neuropathy, they illustrate the principle that IOP-induced strain can alter posterior pole morphology. In the context of myopia development, it is plausible that similar, more diffuse IOP-related strain could contribute to subtle changes in the curvature of the posterior globe, thereby altering peripheral refractive error. The concept of ocular rigidity links biomechanics to fluid dynamics. A study found a strong inverse correlation between pulsatile choroidal volume change and ocular rigidity (21). Since peripheral defocus signals are processed by the retina, which overlies the choroid, changes in choroidal thickness and pulsatility—which are biomechanically influenced by IOP and ocular rigidity—could potentially interact with the retinal processing of defocus. Although no study directly links IOP to peripheral defocus modulation, the biomechanical framework exists. IOP maintains baseline stress on the sclera (17). If the sclera’s material properties are altered during myopic progression—becoming more compliant (23).the same level of IOP could produce a different strain pattern, possibly exaggerating peripheral optical aberrations. In conclusion, while the direct pathway requires further elucidation, IOP perhaps interacts with peripheral retinal defocus through biomechanical means. By influencing the stiffness and shape of the ocular coat, particularly the sclera, IOP can affect the globe’s morphology. This, in turn, it could modulate the quality and characteristics of the peripheral retinal image, potentially amplifying or modifying the defocus signal that guides axial growth. While, this biomechanical-optical coupling needs more research to represent it as a area for understanding myopia pathogenesis.

### 3.3 Intraocular pressure and corneal curvature and higher-order aberrations

There are currently no relevant direct reports on the study of intraocular pressure and corneal curvature and higher-order aberrations. In a study (26) aimed at evaluating preoperative predictors of successful outcomes after corneal stromal ring implantation, the subjects were patients with keratoconus. Intraocular pressure was recorded as one of the preoperative assessment parameters. However, the study results did not report statistically significant associations between intraocular pressure and corneal curvature (such as the mean corneal curvature Km) or higher-order aberrations. Regarding the potential role of intraocular pressure in influencing corneal curvature and higher-order aberrations, and how these factors indirectly affect myopia progression, this literature does not provide directly relevant evidence or analysis.

## 4 Exploring the role of intraocular pressure in the response to myopia interventions

### 4.1 Analysis of the association between low-level red-light therapy and intraocular pressure

A real-world study investigating repeated low-level red-light (RLRL) irradiation for myopia control provides direct evidence regarding the relationship between intraocular pressure (IOP) and therapeutic efficacy. This retrospective case study, which included 323 children and adolescents, systematically analyzed factors correlated with axial length (AL) changes over a follow-up period extending up to 24 months (27). The analysis specifically examined baseline ocular parameters, including IOP, corneal curvature, and corneal thickness, in relation to the degree of AL change. The results demonstrated that the amount of AL change during follow-up showed statistically significant variation, with the most pronounced axial shortening observed at the 6-month follow-up (△AL = -0.16 ± 0.18 mm) (27). Crucially, the study found no statistically significant correlation between the baseline IOP and the subsequent changes in axial length (*p* > 0.05) (27). This finding indicates that within the context of RLRL therapy, the baseline level of IOP is not a predictive factor for the treatment’s effect on controlling axial elongation. This observation is consistent with other studies evaluating RLRL’s impact, which primarily focus on biometric and refractive outcomes without identifying IOP as a mediating variable (28, 29). For instance, a separate retrospective cohort study confirmed that RLRL therapy significantly slowed axial elongation and myopic refractive progression over 12 months compared to a control group using single-vision spectacles, yet reported no significant between-group differences in IOP measurements (29). Another study on adolescents with mild to moderate myopia also reported stable IOP measurements among other secondary outcomes over a six-month RLRL treatment period, with no significant changes observed (28). Collectively, these results suggest that the mechanism by which RLRL therapy exerts its myopia-controlling effect is likely independent of IOP modulation. The primary mechanisms are hypothesized to involve other biological pathways, such as the stimulation of retinal dopamine release, induction of choroidal thickening, and enhancement of choroidal blood flow (30, 31). Studies utilizing swept-source optical coherence tomography angiography (SS-OCTA) have shown that RLRL exposure significantly increases subfoveal choroidal thickness and choroidal vessel volume in pediatric patients, changes that are positively correlated with the reduction in axial length (30). Furthermore, predictors for a good response to RLRL therapy have been identified as longer baseline AL and lower spherical equivalent refraction (SER), with treatment compliance also showing a trend toward significance, while IOP was not highlighted as a predictive factor (31). Therefore, in this specific interventional context, IOP does not appear to function as a key intermediary variable linking the treatment to its anatomical outcome (axial length control), reinforcing the notion that RLRL’s efficacy operates through alternative, IOP-independent neurovascular and structural pathways within the eye.

### 4.2 Significance of intraocular pressure in the context of other combined prevention and control measures

The same extensive real-world study that found no correlation between baseline IOP and axial length (AL) changes also revealed a significant finding regarding combined interventions (27). The analysis indicated that at the 6-month, 12-month, and 18-month follow-up time points, the combination of RLRL therapy with other myopia prevention and control measures was statistically correlated with the change in AL (*p* < 0.05) (27). This suggests that within a multimodal management strategy, the overall efficacy on eye growth may be influenced by the synergistic or additive effects of concurrent interventions. In such a scenario, IOP is not the primary causative factor for change. A systematic review protocol for photobiomodulation therapy (PBMT), which includes RLRL, aims to analyze disease-specific outcomes like IOP in conditions such as glaucoma, highlighting that IOP remains a critical clinical parameter in ophthalmic health assessment across various diseases (32). Therefore, in a combined prevention and control system, monitoring IOP retains clinical importance for overall ocular safety, even if it is not the direct target of RLRL’s action. The study on predictors for a good RLRL response further underscores the complexity of individual factors, identifying baseline AL and spherical equivalent refraction (SER) as key predictors, with treatment compliance being nearly significant, but not IOP (31). This reinforces that the main determinants of RLRL success lie elsewhere. However, when RLRL is paired with other methods—such as specific optical designs, pharmacological agents, or behavioral modifications—the physiological milieu of the eye is altered in a more complex manner. In this integrated context, baseline IOP, as a fundamental biometric parameter reflecting ocular biomechanics and homeostasis, might influence or be influenced by the combined regimen, potentially affecting the final AL outcome indirectly. Consequently, for clinicians designing and evaluating comprehensive myopia management plans, it is prudent to record and consider individual baseline IOP values. This practice ensures a holistic safety profile and helps decipher the contribution of each component within the combined strategy, even though the therapeutic effect of RLRL itself on slowing axial elongation appears to operate through mechanisms largely independent of IOP regulation, primarily involving choroidal thickening and enhanced blood flow (29, 30).

## 5 Association study between intraocular pressure and myopia-related structural changes

### 5.1 Relationship between intraocular pressure and optic disc morphological parameters

In children and adolescents with myopia, optic disc tilt and displacement are common phenomena, and the relationship between these morphological alterations and intraocular pressure (IOP) is a subject of ongoing investigation. A longitudinal study focusing on children with a large vertical cup-to-disc ratio (vCDR) found that progressive optic disc tilt during myopia progression was significantly associated with a higher risk of developing retinal nerve fiber layer defects (RNFLD) (26). While this study did not directly report an independent association between IOP and disc tilt. Research utilizing animal models provides further insights into these relationships. A study in guinea pigs with form-deprivation myopia, which also underwent scleral crosslinking, reported that after adjusting for age, IOP showed a positive correlation with the average cup-to-disc ratio and a negative correlation with the neuroretinal rim area (33). This suggests that even in developing eyes, IOP may influence certain ONH parameters indicative of glaucomatous change. However, the primary driver of disc morphology in myopia appears to be axial elongation. Studies using advanced imaging like ultra-high field MRI have demonstrated that the retrobulbar optic nerve and subarachnoid space dimensions are significantly influenced by the degree of myopia, with longer axial lengths associated with smaller optic nerve areas, independent of Bruch’s membrane opening size (34). This points to the mechanical stretch from eye growth as a dominant factor. The appearance of peripapillary hyper-reflective ovoid mass-like structures (PHOMS) is another morphological feature linked to myopia. Future, more granular studies are needed to disentangle the causal relationships between IOP, the biomechanical forces of axial elongation, optic disc tilt, and the formation of structures like PHOMS. The biomechanical susceptibility of the ONH to pressure may also vary with myopia status. A pilot study investigating ONH pulsatile displacement in primary open-angle glaucoma (POAG) patients found that IOP reduction led to a significant decrease in pulsatile tissue movement in non-myopic eyes, but this effect was not statistically significant in eyes with axial myopia (35). This indicates that the ONH in myopic eyes may respond differently to IOP changes, possibly due to altered scleral compliance or pre-existing structural deformation. Furthermore, research has shown that eyes with high myopia alone exhibit lower ONH strain under acute IOP elevation compared to eyes with pathologic myopia or those with both high myopia and glaucoma, suggesting that the presence of glaucoma or more advanced myopic pathology alters biomechanical susceptibility (36). In summary, while IOP is frequently accounted for in analyses of ONH morphology in young myopes, current evidence suggests that axial length and refractive error are more strongly correlated with parameters like disc tilt (26). The mechanical stretching resulting from axial elongation is the predominant driver of these morphological changes in the developing myopic eye. IOP’s contribution remains speculative and requires further investigation.

### 5.2 Relationship between intraocular pressure and retinal nerve Fiber layer and ganglion cell complex thickness

The assessment of retinal nerve fiber layer (RNFL) and ganglion cell complex (GCC) thickness in children and adolescents with myopia requires careful consideration of ocular magnification effects, and studies employing magnification-corrected imaging, such as swept-source optical coherence tomography (SS-OCT), provide valuable insights. In these investigations, intraocular pressure (IOP) is often included as a covariate in analytical models to evaluate its potential influence alongside other key ocular parameters. However, the prevailing evidence indicates that in this demographic, axial length (AL) and spherical equivalent (SE) refractive error are the predominant factors associated with variations in peripapillary RNFL and macular GCC thickness, with the independent contribution of IOP being relatively modest. This underscores the notion that during the developmental period of myopia progression, the pathological forces of optical defocus and the consequent mechanical stretching from axial elongation are likely the dominant drivers of structural changes in the inner retina and optic nerve head, rather than IOP alone. A foundational study in children with large vertical cup-to-disc ratios demonstrated that a greater change in spherical equivalent (i.e., myopia progression) and a greater increase in optic disc tilt ratio were significantly associated with a higher risk of developing RNFL defects over a long-term follow-up, whereas IOP, while measured, was not highlighted as an independent risk factor in the final model (26). This aligns with research in adult populations and disease models. For instance, a study on guinea pigs with induced myopia found that after adjusting for age, IOP was correlated with ONH structural parameters like cup-to-disc ratio but not specifically isolated as the primary driver of RNFL changes in the context of myopic progression (33). The biomechanical impact of IOP may be context-dependent. Research on POAG patients showed that IOP reduction significantly decreased pulsatile ONH displacement in non-myopic eyes but not in those with axial myopia, suggesting that the relationship between IOP and ONH/retinal tissue mechanics is altered in elongated eyes (35). Furthermore, a study examining ONH strain under acute IOP elevation found that eyes with high myopia and glaucoma experienced greater strain than eyes with high myopia alone, indicating that the presence of glaucoma modifies tissue susceptibility, potentially involving the RNFL and GCC (36). In clinical evaluations of glaucoma suspects, elevated IOP is a well-established risk factor for conversion to primary open-angle glaucoma (37). However, in the specific context of assessing RNFL/GCC thickness in pediatric myopia without overt glaucoma, the signal from axial elongation is stronger. Studies on deep ONH structures in highly myopic eyes with peripapillary intrachoroidal cavitation (PICC) found that worse visual field sensitivity corresponding to the PICC location was associated with the presence of full-thickness retinal defects and circumpapillary RNFL thinning, but not with PICC depth or other myopia-related deep ONH parameters after multivariate adjustment (38). This suggests that local tissue disruption and RNFL loss are key structural correlates of function, with IOP being one of several background factors in the model. Similarly, an analysis of ocular parameters in adults with myopic anisometropia found that the more myopic eye had lower peripapillary RNFL thickness among other alterations, with the interocular refractive difference strongly correlated with interocular AL difference, again emphasizing the role of axial elongation (39). Therefore, while IOP is routinely controlled for in statistical models assessing RNFL and GCC (26). The collective evidence points to AL and SE as the principal determinants of these thickness measurements in children and adolescents with myopia. This reflects the unique pathophysiology of developmental myopia, where the mechanical stress from scleral stretching and remodeling may overshadow the contribution of normative IOP levels to retinal ganglion cell and nerve fiber layer metrics, except in cases where IOP is pathologically elevated or in the presence of comorbid glaucomatous processes.

## 6 Conclusion

The relationship between intraocular pressure (IOP) and myopia development represents a complex and evolving area of research within ophthalmology. While the primary drivers of myopia—axial elongation and scleral remodeling—are well-established, the role of IOP as a potential modulating factor has garnered increasing attention. Current epidemiological evidence suggests a modest association between lower IOP and a reduced prevalence and slower progression of myopia, particularly in specific environmental contexts such as high-altitude regions. However, the strength of this association is notably weaker compared to core biometric parameters like axial length, indicating that IOP is likely a secondary or permissive factor rather than a primary driver.

From a mechanistic standpoint, IOP might be hypothesized to influence myopia progression indirectly through its effects on the biomechanical homeostasis of the posterior sclera. The sclera, as the outer coat of the eye, is subjected to continuous stress from IOP. In the context of myopia, the sclera undergoes significant remodeling, characterized by thinning and reduced collagen density, which may alter its biomechanical response to IOP. It is plausible that IOP interacts with retinal defocus signals, potentially modulating the biochemical cascades that lead to scleral extracellular matrix degradation and axial elongation. For instance, a higher IOP might exacerbate the mechanical strain on a sclera already weakened by myopic remodeling, thereby accelerating elongation. Conversely, a lower IOP might provide a more stable mechanical environment, slowing progression. However, the precise molecular pathways through which IOP exerts these effects, and its relative weight compared to other biochemical signals like transforming growth factor-beta or matrix metalloproteinases, remain largely unelucidated. This gap in knowledge highlights the need for integrated research that combines biomechanics with molecular biology.

The clinical translation of IOP’s role in myopia intervention has been limited. Current first-line therapies, such as low-dose atropine and specialized optical designs (e.g., peripheral defocus correction lenses), primarily target retinal signaling pathways and accommodation mechanisms, with no direct evidence supporting IOP modulation as a key component of their efficacy. Notably, studies on low-level red light therapy, an emerging intervention, have not shown a correlation between IOP changes and therapeutic outcomes, further underscoring that IOP’s direct regulatory role in standard myopia control is minimal. This does not, however, diminish its importance in specific pathological contexts.

Balancing these diverse perspectives—epidemiological associations, indirect biomechanical roles, limited direct interventional impact, and critical importance in pathological states—requires a stratified approach. For the general myopic population, IOP should not be a primary target for intervention given the current evidence. Instead, resources should be focused on proven strategies targeting axial length. However, IOP merits consideration as a potential biomarker for susceptibility or progression rate in certain subgroups, warranting its measurement in comprehensive myopia management protocols as part of a holistic assessment.

Future research must address several key avenues to clarify IOP’s role. First, well-designed prospective cohort studies are essential to establish the temporal sequence and potential causal relationship between IOP fluctuations and axial elongation, moving beyond cross-sectional correlations. Second, the application of advanced computational biomechanical models of the ocular shell can help quantify the stress and strain distributions induced by IOP on the posterior sclera during myopia progression. These models, validated with *in vivo* data, could define a “biomechanical risk profile.” Third, as the field explores novel interventions targeting scleral biomechanics directly—such as collagen cross-linking or specific pharmacologic agents to strengthen the sclera—the interaction of these therapies with IOP must be rigorously investigated. It is within these next-generation strategies that IOP might find a more defined role, either as a parameter to monitor or a factor to co-manage.

In conclusion, while intraocular pressure is unlikely to be a central therapeutic target for halting common myopia progression, it represents a significant piece in the complex puzzle of ocular growth regulation. Its influence appears contextual, acting as a subtle modulator in typical myopia and a potent driver in comorbid pathological conditions. A balanced view acknowledges its potential biomechanical contribution without overstating its clinical utility in standard care. Advancing our understanding will require interdisciplinary efforts, bridging epidemiology, biomechanical engineering, and molecular biology, to ultimately refine risk stratification and inform the development of more personalized and effective myopia control strategies.
